# Interventions Targeting Insulin Resistance in Patients with Type 1 Diabetes: A Narrative Review

**DOI:** 10.3390/medicina60122067

**Published:** 2024-12-16

**Authors:** Andreea Herascu, Vlad-Florian Avram, Laura Gaita, Sima Alexandra, Delia-Viola Reurean-Pintilei, Bogdan Timar

**Affiliations:** 1 Doctoral School of Medicine, “Victor Babes” University of Medicine and Pharmacy, 300041 Timisoara, Romania; andreea.herascu@umft.ro; 2 Department of Diabetes, “Pius Brinzeu” Emergency Hospital, 300723 Timisoara, Romania; gaita.laura@umft.ro (L.G.); sima.alexandra@umft.ro (S.A.); bogdan.timar@umft.ro (B.T.); 3 Centre for Molecular Research in Nephrology and Vascular Disease, “Victor Babes” University of Medicine and Pharmacy, 300041 Timisoara, Romania; 4 Second Department of Internal Medicine, “Victor Babes” University of Medicine and Pharmacy, 300041 Timisoara, Romania; 5 Department of Medical-Surgical and Complementary Sciences, Faculty of Medicine and Biological Sciences, “Stefan cel Mare” University, 720229 Suceava, Romania; delia.pintilei@usm.ro; 6 Department of Diabetes, Nutrition and Metabolic Diseases, Consultmed Medical Centre, 700544 Iasi, Romania

**Keywords:** insulin resistance, diabetes mellitus, targeting interventions

## Abstract

*Background and Objectives:* Insulin resistance (IR) is the most important factor involved in the pathogenesis of type 2 diabetes but may also develop in type 1 diabetes (T1DM). Developing IR in patients with T1DM may generate a burden in achieving glycemic targets and may deteriorate the overall prognosis. This review aims to describe the pathogenesis of IR in T1DM, summarize the common associations of IR with other conditions in patients with T1DM, describe the consequences of developing IR in these patients, and present the interventions that target IR in people with T1DM. *Results:* The occurrence of IR in T1DM is multifactorial; however, it is frequently linked to overweight or obesity and sedentary lifestyle. Besides impairments in glycemic control and increased insulin requirements, the presence of IR is associated with an increased cardiovascular risk in patients with T1DM. Considering that patients with T1DM are insulin-treated, IR may be evaluated only using surrogate biomarkers, the most frequently used being the estimated glucose disposal rate. The most important interventions that are shown to be feasible in improving insulin sensitivity in patients with T1DM are lifestyle optimizations, including nutrition therapy or physical activity and pharmacotherapy with metformin, sodium-glucose cotransporter-2 inhibitors, glucagon-like peptide-1 receptor agonists, and thiazolidinediones. *Conclusions:* Targeting the improvement of IR in patients with T1DM is a key element in achieving optimal glycemic control, as well as improving the overall patient’s prognosis besides glycemic control.

## 1. Background and Objectives

### 1.1. Diabetes—A Global Public Health Issue

Diabetes currently represents a major global public health issue, as it plays a major role in determining morbidity and mortality. According to the report made by IDF in 2021, an estimated 537 million people suffer from this disease worldwide [1]. By the year 2045, it is expected that this number will increase to approximately 783 million cases of diabetes. The worldwide financial burden of diabetes on the world’s healthcare systems amounts to roughly USD 966 billion [2].

### 1.2. Type 1 Diabetes vs. Type 2 Diabetes

There are two major types of diabetes, together representing more than 95% of the total cases: type 1 diabetes, determined by an absolute insulin deficiency, and type 2 diabetes, characterized by insulin resistance, which is associated with a relative dysfunction in insulin production or function [3].

Type 1 diabetes mellitus (T1DM) is an autoimmune disease, and it is characterized by the destruction of the insulin-producing pancreatic β-cells, mediated by T-lymphocytes. The occurrence of T1DM is promoted by an interaction between an individual’s genetics and the environment. The interaction between genetic factors (HLA class molecules, such as DR4, DQ8, and DQ2, are present in 90% of T1DM) and environmental factors promote the recognition of the betta cell as autoantigens, which are erroneously targeted by the immune system, triggering the autoimmune response [4].

Insulin resistance represents the main pathogenic driver for T2DM and is characterized as a low response of insulin-targeting tissues to normal levels of insulin. Insulin resistance precedes elevated plasma glucose levels, which are non-physiologic, and this state represents the main clinical symptom of T2DM. In the prediabetic state, insulinemia increases in order to meet normal insulin requirements, which leads to chronic hyperinsulinemia and hyperglycemia caused by β-cell failure, and the final stage is represented by the appearance of T2DM [5].

While type 2 diabetes is generally thought of as the insulin-resistant type of diabetes, it can be mentioned that insulin resistance may also be present in T1DM as well [6]. The presence of obesity and a sedentary lifestyle are well-known factors leading to increased insulin resistance, and T1DM patients are not exempt from these factors. As such, insulin resistance has been proposed as a possible factor that may lead to improper glucose control in patients with T1DM [6,7].

### 1.3. Insulin Resistance

Glucose homeostasis is conditioned by a normal insulin secretory response and a normal tissue sensitivity to increased glucose uptake. There are three tightly coupled mechanisms that enhance glucose disposal: suppression of endogenous glucose production and increasing glucose uptake by hepatic and peripheral tissues (mainly muscle). The mechanisms involved in the pathogenesis of T2DM are complex and involve various elements that, when added together, lead to increases in blood glucose levels. Insulin resistance is characterized by decreased peripheral glucose uptake (especially in muscle) and increased endogenous glucose production. All tissues that have insulin receptors can be insulin-resistant, but the main tissues that become insulin-resistant are the liver, the adipose tissue, and the skeletal muscle. This results in an increase in β-cell insulin production, which leads to hyperinsulinemia. Lipolysis, increased free fatty acid levels, and accumulation of intermediary lipid metabolites increase glucose output, lower peripheral glucose consumption, and affect β-cell function [8,9].

Inflammation and adipocyte insulin resistance have been shown to be important components of the development of T2DM. Once the levels of inflammatory cytokines become more prominent, insulin resistance also increases. While the latter also influences the former in the same manner, it is generally accepted that inflammation is a major driver of insulin resistance [10,11].

While an initial compensatory increase in insulin secretion by the pancreatic β-cells initially may maintain normal glucose levels, this effect is not lasting as β-cell function is already altered at this stage, and it will deteriorate over time. Furthermore, there is an inadequate release of glucagon, especially in the postprandial period, from the pancreatic α-cells, additionally altering insulin secretion signals. Impaired insulin and also excessive glucagon secretion are derived from an “incretin defect”, which is defined by deficient release or response of the incretin hormones after a meal [8].

During the fasting state, normally, the liver produces glucose to maintain a normal glycemia level and provide a supply for glucose-consuming tissues. For this process to be possible, the liver either breaks down glycogen (glycogenolysis) or synthesizes new glucose molecules (gluconeogenesis) by using fatty acids and glycerol. During the postprandial periods, β-cells secrete insulin, which promotes anabolism while also suppressing catabolic processes. Insulin prompts glucose-consuming tissues, such as adipose tissue and skeletal muscle, to uptake blood glucose and convert it into glycogen and lipids. Other insulin-mediated effects are to suppress hepatic gluconeogenesis and glucagon secretion from α-cells and to decrease appetite via the central nervous system [5,7]. Also, insulin increases glucose uptake and glycolysis, which indirectly increases glucose oxidation [12].

As mentioned above, in the insulin resistance phase, higher-than-normal levels of insulin are required in order to maintain the normal function of this hormone. Since skeletal muscle represents the quantitatively central tissue for insulin-mediated glucose disposal, and adipose tissue and liver represent key sites for glucose uptake mediated by insulin, these tissues are considered to be of paramount importance in the mechanism involved in the emergence of insulin resistance [5,11].

The appearance of insulin resistance usually consists of impaired glucose disposal at the level of insulin-resistant tissues, primarily skeletal muscle [13]. As a result, considering the excess calorie consumption, more insulin is needed to transport glucose into these tissues. This vicious cycle continues until β-cell activity can no longer meet the insulin demand in an adequate way, resulting in hyperglycemia. With a continued discrepancy between insulin requirement and insulin production, there is a rise in glycemic levels up to those consistent with type 2 diabetes [14]. Usually, hyperinsulinemia is associated with weight gain but may also be related to a chronic calorie excess. As tissues become more insulin-resistant, the anabolic effect of this hormone decreases, and weight gain finally slows. Resistance to exogenous insulin is also described. A clinical benchmark defines a patient as insulin-resistant if more than 1 unit/kg/day of exogenously administrated insulin is required and daily needs exceeding 200 units per day are considered severe insulin resistance [9].

Muscles are the main site for glucose disposal after caloric intake, comprising up to 70% of tissue glucose consumption. In chronic caloric excess, skeletal muscle tissue stores up intramyocellular fatty acids. Diacylglycerol represents an intramyocellular fatty acid that indicates energy excess inside the cell. Consecutively, protein kinase C theta is activated, and insulin signaling is decreased. The result is reduced glucose intake by the skeletal muscle tissue due to decreased GLUT4 translocation to the cell membrane. The excess glucose is transported to the liver to be metabolized or stored [15].

The liver is considered to be responsible for maneuvering energy substrates. It processes, stores, and creates glucose and packages, recirculates, and generates fatty acids [16]. These processes are significantly disrupted when the liver reaches the insulin-resistant state, leading to important metabolic consequences. De novo lipogenesis using excess glucose from the blood and fatty acids from adipose tissue lipolysis represents an important contributor to hepatic VLDL secretion [15].

Research has determined that the lipolysis process is sensitive to insulin, so the increase in circulating free fatty acids especially occurs when insulin fails to inhibit lipolysis at the level of insulin-resistant adipose tissue, particularly visceral adipose tissue [17].

**Figure 1:**
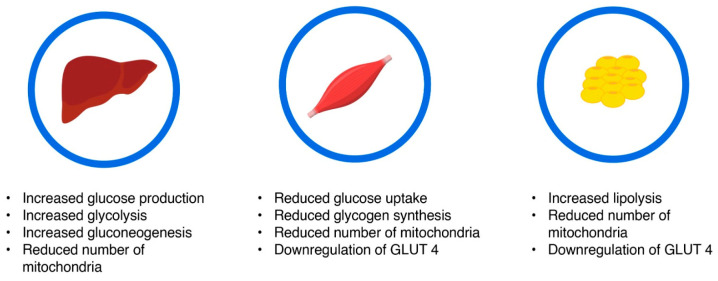
Main characteristics of insulin resistance. Insulin resistant states are characterized by increased hepatic glucose production, decreased uptake of insulin in peripheral tissues, and a downregulation of GLUT transporters. GLUT-Glucose transporter.

Insulin resistance is mainly promoted by excess body fat, although genetic factors also play a role. In the absence of a generally accepted test for insulin resistance, the clinical definition remains blurred. Clinically, insulin resistance is assessed through the metabolic consequences associated with this state and can be modified by exercise [18]. Main characteristics of insulin resistance have been summarized in Figure 1.

A more recently discussed mechanism in the development of insulin resistance is mitochondrial dysfunction [19]. Insulin secretion in pancreatic β-cells is dependent on ATP production. As such, in the presence of high insulin resistance, when insulin levels increase, the need for ATP also rises. The increase in ATP production leads to increases in reactive oxygen species, to which pancreatic β-cells are highly sensitive. This prompts the activation of uncoupling proteins in order to reduce the production of reactive oxygen species and, in turn, that of ATP, blocking insulin secretion [20].

Patients with DM have been shown to display a decreased number of mitochondria per cell and, therefore, present impaired ATP production, which, in turn, leads to impaired insulin production in β-cells over time [19,20,21]. Furthermore, the lack of ATP in muscle and adipose tissue cells leads to impaired glucose uptake due to the fact that insulin receptors function via tyrosine kinase and, consequently, are dependent on the presence of ATP [19,22,23].

Insulin binds to the insulin receptor of cells, triggering a chain reaction via insulin receptor substrates (IRS-1 and IRS-2), leading to activation of TC10 by C3G, which, in turn, determines the translocation of insulin-dependent glucose transporters (GLUT 4) found in the cytoplasm of cells to the cells’ membrane in order to facilitate glucose uptake in the cell. There are several types of glucose transporters, each being associated with a certain cell type. The translocation of insulin-dependent glucose transporters (GLUT4) found in the cytoplasm of skeletal muscle myocytes and adipocytes is downregulated by chronic hyperglycemia [24,25].

Some studies suggest that this defect appears to be due to the ability of fatty acids to inhibit insulin-stimulated glucose transport at the skeletal muscle (GLUT4). Increased fatty acid delivery to the liver and muscle may be due to several mechanisms, including excess energy intake, defects in adipocyte fat metabolism, or acquired/inherited defects in mitochondrial fatty acid oxidation [26].

**Figure 2:**
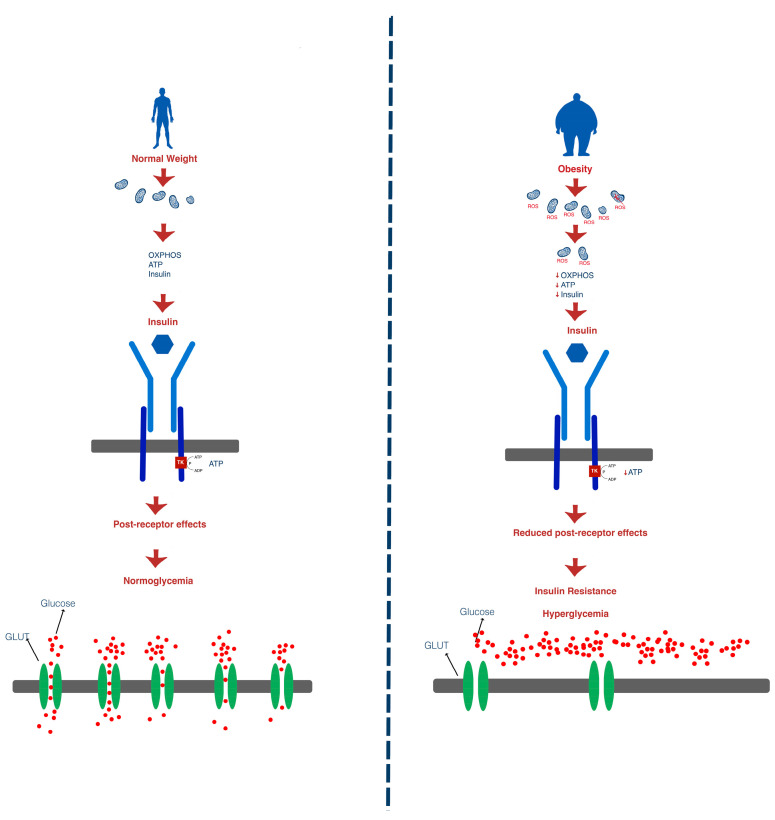
Metabolic modifications in energy metabolism leading to insulin resistance and hyperglycemia. The reduction in mitochondrial count in the insulin-resistant state leads to a reduction in ATP production, leaving the insulin receptor’s tyrosine kinase domain with impaired function. This leads to a reduction in post-receptor response and downregulation of GLUT transporters, which results in impaired glucose uptake and hyperglycemia. ROS—reactive oxygen species; OXPHOS—oxidative phosphorylation; ATP—adenosine triphosphate; ADP—fadenosine diphosphate; TK—Tyrosine kinases; GLUT—Glucose transporter.

The gold standard measurement for insulin resistance is represented by the hyperinsulinemic–euglycemic glucose clamp technique, but this procedure has limited clinical applicability. In order to suppress hepatic glucose production, a fasting, non-diabetic patient is put on a high-rate constant infusion of insulin. Blood glucose is regularly monitored, and a concurrent 20% dextrose solution is administered at different rates to keep the blood glucose within the euglycemic range. The exogenous glucose elimination required to make up for hyperinsulinemia is reflected in the amount of glucose needed to achieve a steady state. Body size and whole-body glucose disposal are used to calculate insulin resistance. However, there are surrogate measures of insulin resistance that are useful in clinical practice. One commonly used indicator of insulin resistance in clinical research is the homeostatic model assessment for insulin resistance (HOMA-IR), which is based on fasting glucose and fasting insulin levels. When defining insulin resistance, the threshold HOMA-IR values exhibit significant variability. There are variations in HOMA-IR levels by age and gender, with higher levels in women over fifty. The threshold HOMA-IR levels used to characterize insulin resistance vary widely. In various geographical locations, population-based studies have been conducted in order to determine the cut-off values of HOMA-IR for the diagnosis of insulin resistance [27]. There are other measures that are based on fasting insulin, such as homeostasis model assessment 2 (HOMA2), quantitative insulin sensitivity check index (QUICKI), and glucose infusion rate (GIR). Oral glucose tolerance test, realized after overnight fasting, measures insulin and glucose response after the ingestion of 75 g glucose. Other markers for insulin resistance are serum triglyceride and triglyceride/HDL ratio. The triglyceride/HDL ratio indicates average cut-offs of 2.53 for women and 2.8 for men, and this is valid for both children and adults of various weights and ages; however, more research is needed on customized cut-offs that account for gender and ethnic variations [28]. Patients with prediabetes and triglycerides over or equal to 150 g/dL commonly presented insulin resistance [9,29,30,31]. The metabolic modifications in energy metabolism leading to insulin resistance and hyperglycemia have been summarized in Figure 2.

The pancreas releases more insulin in response to the body’s insulin resistance in order to maintain cell energy and control blood glucose levels within a healthy range. This explains the fact that circulating insulin levels are often higher in the early phases of type 2 diabetes. Since the pancreas may produce more insulin, insulin resistance by itself will not initially cause any symptoms. However, insulin resistance tends to worsen over time, and beta cells’ insulin production may gradually deteriorate. The pancreas is unable to produce enough insulin to overcome insulin resistance. Higher blood glucose levels and, eventually, prediabetes or type 2 diabetes are the outcomes [32].

Insulin is secreted by the pancreas during hyperglycemia in a biphasic manner. The first phase, consisting of preformed insulin, lasts approximately 10 min and is the first to be affected in insulin-resistant individuals. The second phase of insulin secretion persists for the duration of hyperglycemia and is also altered; however, it is not to the same extent as the first phase in the initial stages of the disease. Chronic hyperglycemia and disease progression lead to a dysfunctional secretion of the β-cells, lowering insulin secretion in both phases [33].

### 1.4. Consequences of Insulin Resistance

Insulin resistance is a primary risk factor for numerous medical conditions and is a major characteristic of metabolic disorders, which are believed to be the pathogenic cause of many contemporary diseases. Additionally, it is generally acknowledged that IR has a role in the etiology of the majority of metabolic disorders, atherosclerosis, cardiovascular disease (CVD), several types of cancer, and neurodegenerative illnesses. As a result, IR is thought to be the main cause of numerous illnesses and plays a major role in the epidemic of chronic diseases [34]. Metabolic syndrome is a collection of metabolic traits, which includes obesity, dyslipidemia, hypertension, and insulin resistance. It has also been connected to an increased risk of heart disease and type 2 diabetes [18]. Both an increase in body fat and a tendency for excessive accumulation of fat in the upper body are linked to insulin resistance. Overweight women with a higher body fat percentage in the chest and abdomen have higher levels of insulin resistance, glucose intolerance, dyslipidemia, and hyperinsulinism than women who are overweight but have a lower percentage of body fat. Therefore, one of the key components of metabolic syndrome is the percentage of body fat [34].

#### 1.4.1. Obesity

T1DM and obesity have a more complicated and not as straightforward link than obesity and T2DM [35].The excessive accumulation of body fat in individuals with type 1 diabetes is thought to be caused by non-physiological insulin replacement that results in peripheral hyperinsulinemia, insulin profiles that do not match basal and mealtime insulin needs, defensive snacking to prevent hypoglycemia, or a combination of these [36].

Obesity has almost tripled in incidence worldwide since the 1970s, and it is now considered an epidemic [10]. Obesity plays a major role in heart disease, hypertension, dyslipidemia, type 2 diabetes, sleep apnea, and several types of cancer. This makes it one of the most dangerous health problems, even though it is largely preventable [34]. There is a correlation between peripheral insulin resistance and obesity, particularly abdominal obesity. Within obesity, insulin resistance varies widely, and higher levels of insulin resistance are associated with the highest risk of heart disease and T2DM. The majority of patients with insulin resistance are overweight or obese, even though obesity and insulin resistance are not always related [37].

#### 1.4.2. Diabetes Mellitus

The primary consequence of insulin resistance is T2DM. It is thought that insulin resistance precedes the appearance of T2DM by 10–15 years, although recent studies discussed whether hyperinsulinemia paves the way for insulin resistance; in other words, if hyperinsulinemia itself represents a driver of insulin resistance. The metabolic effects of insulin resistance consist of hyperglycemia, hypertension, elevated inflammatory markers, endothelial dysfunction, prothrombotic state, dyslipidemia, and hyperuricemia. Progression of insulin resistance may lead to T2DM, metabolically associated fatty liver disease, and metabolic syndrome. In addition to T2DM, insulin resistance is also associated with obesity, metabolic syndrome, microvascular disease (retinopathy, nephropathy, and neuropathy), macrovascular disease (diabetic heart disease, stroke, and peripheral artery disease), metabolically associated fatty liver disease, and polycystic ovary syndrome [8,22].

Insulin resistance in the primary insulin target tissues is a recognized abnormality that occurs before T2DM develops [38]. An important characteristic of the metabolic syndrome is hyperinsulinemia, which is brought on by excessive β-cell insulin production and has been connected to the development of T2DM and cardiovascular disease [39].

#### 1.4.3. Non-Alcoholic Fatty Liver Disease

The pathophysiology of non-alcoholic fatty liver disease and its progression from steatosis to steatohepatitis, cirrhosis, and, ultimately, hepatocellular carcinoma—noted to be more prevalent in those with T2DM—are both linked to insulin resistance [40]. Insulin-resistant, dysfunctional adipose tissue disrupts this crucial glucose–lipid (FFA) energy transition, causing metabolic inflexibility, a hallmark of insulin-resistant states such as obesity, metabolically associated fatty liver disease (MAFLD), and type 2 diabetes. At the expense of glucose consumption, this disruption encourages a persistent, chronic excess of fat (FFAs) as the primary daily source of energy for the muscle and liver. Lipotoxicity is the term for this long-term FFA surplus energy source that is characteristic of insulin-resistant conditions [41]. Hepatic steatosis is triggered by a combination of elevated hepatic DNL rates, compromised glucose uptake in cells in response to insulin, and an excess of energy originating from FFAs via enhanced lipolysis of white adipose tissue [42].

#### 1.4.4. Polycystic Ovary Syndrome

The most frequent disorder affecting women of reproductive age is polycystic ovarian syndrome, a multifactorial illness characterized by excessive luteinizing hormone levels, excess androgen, and anovulation. The role of insulin resistance as a primary etiologic factor has remained undeniable and acknowledged despite the several theories proposed to identify the pathogenic cause of polycystic ovary syndrome. Polycystic ovary syndrome is caused by increased ovarian androgen production stimulated by hyperinsulinemia [43], and afflicted women are more likely to suffer T2DM and glucose tolerance disorders [44]. This condition is linked to insulin resistance. Between 60 and 70 percent of women with this syndrome are affected by insulin resistance [45]. Despite being commonly associated with obesity, insulin resistance is just as common in polycystic ovary syndrome women who are not overweight [46].

#### 1.4.5. Cardiovascular Disease

There are multiple molecular processes that connect insulin resistance to cardiovascular disease, including the role of insulin resistance in the development of atherosclerosis, hypertension, and macrophage accumulation. Furthermore, inflammation, insulin resistance, and hyperglycemia are both causal and predictive of harmful cardiovascular events [47]. The development of cardiovascular disease is influenced by the triad consisting of insulin resistance, obesity, and abnormal lipid levels. Additionally, those with a BMI of 30 kg/m^2^ or higher are more prone to develop cardiovascular disease than those with a normal BMI [47]. The fluctuation of fat levels and adipose tissue distribution may have an impact on insulin resistance and cardiometabolic disturbances. About 50% to 70% of the ATP required for cardiac fuel is produced by fatty acid oxidation, whereas glycolysis only accounts for 10% of total ATP synthesis. Although fatty acids seem to be the main energy source, the heart can use alternative ATP sources depending on availability. However, insulin resistance reduces metabolic adaptability, imposing fatty acids as the main energy source. This alteration causes an increase in lipid absorption and accumulation in the heart, which consecutively leads to lipotoxicity [47].

A proatherogenic phenotype is commonly produced by aberrant lipid metabolism brought on by improper insulin signaling, particularly in peripheral tissues like adipose cells. Circulating free fatty acid levels rise long before impaired glucose metabolism occurs in individuals with insulin resistance. Adipocytes may not store fatty acids properly, and lipolysis suppression is lost when insulin signaling is compromised [48]. Due to the post-translational stabilization of apoB, the primary apolipoprotein of VLDL, the excess supply of lipids from various sources (circulating free fatty acids originating in fat, endocytosis of triglyceride-rich lipoproteins, and de novo lipogenesis) improves the assembly and secretion of VLDL particles [49]. VLDL is converted to LDL and residual lipoproteins, which are both highly linked to the risk of atherosclerosis. Triglyceride-rich VLDL particle concentrations are elevated in insulin resistance, which leads to aberrant HDL metabolism [49]. The direct entry of atherogenic VLDL-derived particles into the vasculature or the reduced ability of HDL particles to take part in reverse cholesterol transport, which involves the unloading of cholesterol from the vasculature, may, therefore, cause accelerated atherosclerosis in the context of insulin resistance [50].

Pathways on how insulin resistance, hyperinsulinemia, and the pathophysiology of its clinical consequences contribute to the development of atherosclerosis have been discussed, as there are direct and indirect effects on the artery wall, and strong evidence indicates that insulin targets the endothelium physiologically, suggesting a connection between insulin resistance and atherosclerosis [51]. By increasing the generation of procoagulant mediators and impairing the production of nitric oxide, insulin resistance causes endothelial cell dysfunction and platelet buildup [47]. Atherosclerosis is the leading cause of death in individuals with insulin resistance. Additionally, the atherosclerotic process is regulated by inflammatory pathways. Similarly, insulin resistance was found to be defined as a low-level chronic inflammatory disease [51].

#### 1.4.6. Hypertension

Blood pressure is higher in patients with insulin resistance than in those with normal insulin levels. It is believed that insulin resistance and hyperinsulinemia are significant contributors to hypertension [52]. In the insulin-resistant state, hyperglycemia facilitates intra to extracellular fluid dynamics, which raises blood pressure and plasma volume. Additionally, elevated insulin secretion promotes renin excretion and sodium reabsorption from kidney tubules, as well as sympathetic nerve activation, all of which can lead to a persistent rise in blood pressure [53].

## 2. Insulin Resistance in T1DM

Due to the coexistence of both insulin resistance and the loss of endogenous insulin production, insulin resistance in T1DM is frequently referred to as “double diabetes” [54]. It is now evident that the development of insulin resistance in type 1 diabetes differs from that seen in other metabolic disease states [55]. The phenotypes of T1DM patients who develop insulin resistance and those with T2DM and metabolic syndrome are frequently different. It has been demonstrated that, in comparison to healthy controls, insulin resistance is present in lean, normal body mass index (BMI) individuals with type 1 diabetes [56]. Insulin resistance is also seen in young adults and adolescents with type 1 diabetes who are otherwise healthy and of normal weight. It is linked to poor cardiopulmonary fitness, which includes decreased peak oxygen consumption and oxygen uptake, as well as decreased cardiovascular function, such as left ventricular hypertrophy and diastolic dysfunction [55]. Insulin resistance development in this population cannot be fully explained by hyperglycemia because many T1DM patients continue to have insulin resistance even when their glycemia is managed with aggressive insulin therapy [56]. Typical prognostic markers, such as BMI, visceral adiposity, plasma lipids, physical activity level, glycemic management, and acute glucotoxicity, do not explain the occurrence of insulin resistance in T1DM. In contrast to other insulin-insensitive states, the unique development of IR in T1DM patients points to similar but different mechanisms controlling glucose intolerance in this population. Furthermore, it was demonstrated that IR in T1DM is tissue-specific, mostly appearing in patients’ skeletal muscle and liver [57].

Insulin resistance, a condition that is typically seen in T2DM, is also exhibited by individuals with T1DM. Multiple risk factors may contribute to the development of insulin resistance in T1DM patients, such as genetic predisposition, overweight and obesity, duration of T1DM, oxidative stress, glucotoxicity and lipotoxicity, hormonal changes, age, sex, and ethnic group. The intricate relationships between a person’s genetics and lifestyle are thought to play a significant role in the onset of insulin resistance in T1DM. Insulin resistance is frequently linked to being overweight or obese. This is frequently related to poor glycemic control, elevated insulin dosage needs, and insulin resistance. Overweight and obesity can exacerbate over the course of a person’s life due to aggressive insulin treatment, excessive insulinization, frequent hypoglycemia, and defensive snacking [58].

Consequently, non-pharmacological strategies that can prevent the development of obesity in people with type 1 diabetes have been prioritized [59,60].

Also, insulin resistance in T1DM may be related to how therapeutic exogenous insulin is administered. When compared to normal physiology, insulin absorbed via subcutaneous depots causes hepatic hypoinsulinemia and relative peripheral hyperinsulinemia. Long-term adaptation to this combination may boost hepatic glucose synthesis and decrease peripheral insulin-mediated glucose absorption. Furthermore, it has been suggested that decreased exposure to hepatic insulin lowers the amount of IGF-1 in the blood, which may also lead to an increase in peripheral insulin resistance when combined with a concurrent rise in growth hormone and IGF-binding proteins [61,62]. A validated biomarker of insulin resistance in T1DM is the estimated glucose disposal rate (eGDR), which is derived from data from the hyperinsulinemic–euglycemic clamp method. Common predictor factors in clinical practice, such as BMI (HbA1c) and the presence of arterial hypertension, are used in the eGDR calculation. A common indicator of insulin resistance is eGDR < 8 mg/kg/min [63].

A series of studies have investigated the impact of insulin resistance using eGDR in patients with T1DM and have shown the association between this tool and the prevalence of micro- and macrovascular complications.

A cross-sectional study that included 207 white, black, or Hispanic persons with a history of clinically diagnosed type 1 diabetes who were undergoing treatment at an urban academic medical center was conducted, and eGDR (milligrams per kilogram per minute) was computed. This study has shown that compared to white or Hispanic people, black people had reduced insulin sensitivity, and diabetes complications were associated with lower eGDR [64].

Another cross-sectional study sought to evaluate the level of agreement between the traditional eGDR formula and a different one (eGDR-GMI) that incorporates the glucose management indicator (GMI) obtained from continuous glucose monitoring (CGM) and also investigated the extent of how the prevalence of complications from diabetes cardiovascular risk factors and eGDR-GMI relate to one another. The analysis revealed that eGDR and eGDR-GMI have strong concordance. The study cohort was split into eGDR-GMI tertiles, and the lowest eGDR-GMI tertile had a less favorable metabolic profile and a higher prevalence of diabetes-related comorbidities. For every unit decrease in eGDR-GMI, the relative risk of neuropathy, nephropathy, and retinopathy increased considerably, independent of smoking, lipids, age, sex, and the duration of the disease [65].

Another cross-sectional study, conducted between 2016 and 2020, included 165 consecutive-case patients with T1DM who had no cardiovascular, ocular, or renal complications without further enrollment criteria. Global longitudinal strain and pulse wave velocity were employed as stand-ins for left ventricular systolic dysfunction and subclinical atherosclerosis, respectively. Subclinical cardiac autonomic neuropathy was assessed using four previously standardized procedures that were based on the computation of heart rate variability. Urinary albumin to creatinine ratio was used to evaluate early nephropathy. Insulin resistance, as assessed by eGDR, had a strong correlation with cardiac autonomic neuropathy and early cardiovascular disease predictors in T1D patients. These correlations seem to be unaffected by the effects of age, gender, or disease duration [66].

Another study evaluated the lipoprotein profile, insulin dosage, and estimated glucose disposal rate in overweight or obese children with T1DM in comparison to those having T1DM and normal weight. A number of 115 T1DM patients (ages 5–16) receiving rigorous insulin therapy were enrolled. Weight, height, BMI, hip and waist circumferences, insulin dosage, eGDR, blood pressure, HbA1c, and lipoprotein profile were all measured. Obese children with type 1 diabetes older than 11 years had decreased eGDR readings, which could be interpreted as an indicator of insulin resistance. Patients with diabetes who were overweight or obese received higher doses of insulin, particularly in IU/m^2^/day. Obesity in children with T1DM was more frequently linked to a lipoprotein profile associated with increased cardiovascular risk [67].

Another cross-sectional study aimed to measure insulin resistance in individuals with T1DM using the estimated glucose disposal rate (eGDR) based on whether the metabolic syndrome is present or not and how it relates to long-term complications and concluded that microvascular alterations are linked to insulin resistance, which is prevalent in individuals with T1DM [68].

To predict cardiovascular risk using eGDR, several studies have been conducted. It is widely acknowledged that in people with T1DM, intensive insulin therapy lowers the incidence of CVD. The Diabetes Control and Complications Trial (DCCT), which involved 1441 adolescents and young adults (mean age 27 years, BMI 28 kg/m^2^) randomly assigned to intensive or conventional glycemic control for 6.5 years and followed up in the Epidemiology of Diabetes Interventions and Complications (EDIC) study for a total of 17 years, provides the strongest interventional clinical trial evidence in this matter. A 57% decrease in RR in a composite endpoint of non-fatal MI, stroke, or CV mortality (about six occurrences per 1000 patient years) was linked to intensive glycemic management [69]. Weight gain is undoubtedly a side effect of intensive therapy; during the course of the DCCT research, the group receiving intensive treatment gained 4.6 kg more than the group receiving standard treatment [70].

In this regard, subgroup analyses of the DCCT/EDIC study brought up worries concerning trial participants whose weight gain was linked to the development of characteristics connected to elevated cardiovascular risk. Higher blood pressure, LDL cholesterol, waist-to-hip ratio, a more atherogenic lipid profile (greater VLDL, small, dense LDL, lower apolipoprotein A-I, and lower HDL), and elevated apolipoprotein B were all observed among those in the top quartile for weight gain. The mean BMI in the intensive insulin therapy group increased from 24 to 31 kg/m^2^. These observations point out that despite increases in some biomarkers of cardiovascular risk, like body weight, the risk reductions associated with improvements in glycemic control may counterbalance the overall risk, thus leading to a neutral risk balance in the study [71].

Insulin resistance may affect systems involved in satiety. In a double-blind, placebo-controlled study, Arafat et al. investigated the neuroendocrine stimulation of appetite by giving glucagon intramuscularly to people who were lean (without diabetes mellitus), obese (without diabetes mellitus), or normal weight with T1DM. They then repeated measurements of ghrelin and satiety for 240 min [72]. The orexigenic peptide ghrelin affects a variety of systems, including the central nervous system, increasing the expression of agouti-related peptide from the arcuate nucleus and neuropeptide Y to promote hunger [73]. It is believed that acyl-ghrelin is the active form of ghrelin that causes satiation. Following glucagon administration, Ghrelin dropped in all participants in this trial, but satiety effects persisted in lean people with and without T1DM but not in obese, non-diabetic people [72]. The effects of acyl-ghrelin ranged from a notable drop in lean people to a decrease in people with T1DM, while obese people showed no change. It seems that glucagon control of satiety occurs independently of insulin release. Obese people with type 1 diabetes may gain extra weight due to ghrelin dysregulation and satiety increases [72].

According to a 2014 Danish study including over 75,000 women, obesity raised the likelihood of developing any autoimmune disease, particularly T1DM [74]. Versini et al. carried out a thorough analysis of 329 publications that provide information about the connection between adipokines, obesity, and immune-mediated diseases, including T1DM. Over the course of a person’s life, obesity increases the chance of T1DM. In both human and mouse models, the risk of developing type 1 diabetes is enhanced by higher infant birthweight (particularly > 4000 g) and increased weight gain throughout the early years of life. In pooled investigations, childhood and adolescent obesity doubles the chance of getting T1DM and causes a diagnosis at a younger age. The adipokine profile present in obesity (low adiponectin and high leptin and resistin) promotes the destruction of beta cells [75].

The association between obesity and β-cell autoimmunity was assessed in a prospective cross-sectional study involving 295 pediatric patients with newly diagnosed T1DM. A waist-to-height ratio of 0.5 or higher indicated that 30% of people were centrally obese; these people were typically young (1–4 years old) or in late adolescence (15–18 years old). When comparing centrally obese people to non-centrally obese people, the former showed a worse cardiometabolic profile. Age, type of β-cell autoimmune antibodies, and number of β-cell autoimmune antibodies did not correlate with obesity [76].

Studies relating to insulin-resistant states in T1DM have been summarized in Table 1.

## 3. Results: Interventions Targeting Insulin Resistance

### 3.1. Dietary Interventions

According to some research, people with T1DM consume more saturated fat than people without the disease. Insulin resistance, coronary artery disease, and dyslipidemia are all influenced by a high-fat diet. Saturated fats, low monounsaturated fatty acids, docosahexanoic acid, linoleic acid, and ecosapentanoic acid were found to be taken in greater quantities by patients with low HDL cholesterol or raised triglyceride levels in a study of teenagers with T1DM [77]. Vegetables, whole grains, fruits, and low-fat diets are all advised by the International Society for Pediatric and Adolescent Diabetes (ISPAD) [78]. It has been demonstrated that dietary adjustments increase insulin sensitivity even when there are no changes in glycemic control or body weight [79]. The best outcomes, however, come from a diet that is lower in trans fats and carbs and higher in unsaturated fatty acids. Reducing body weight, consuming fewer simple carbs, avoiding insulin underdosing, substituting saturated fatty acids for polyunsaturated fatty acids, and engaging in regular exercise are all necessary to lower triglycerides [80]. Omega-3 fatty acid ingestion has been demonstrated to improve inflammatory markers. The effect of omega-3 has been explained by a number of theories, such as increased adiponectin levels, the suppression of proinflammatory cytokines, and the expression of nuclear factor-kB (NF-κB) protein. As previously indicated, these compounds can have anti-diabetic benefits due to enhanced insulin metabolism and the anti-inflammatory and anti-atherosclerotic effects caused by inflammation resolution [81]. It is proven that nutrition is a significant modifiable risk factor for the development of insulin resistance in individuals with type 1 diabetes and that a diet rich in protein, low in fat, and optimal in carbohydrates may improve insulin resistance. It is also concluded that a higher dietary fiber intake may lessen the burden of double diabetes by preventing the development of insulin resistance in those with T1DM. A low-fat diet has been shown in another trial to increase peripheral insulin sensitivity in patients with type 1 diabetes [82,83].

### 3.2. Physical Activity

For individuals with T1DM of all ages, physical activity is linked to numerous proven health advantages, such as increased psychological well-being, cardiovascular fitness, and better bone health [84]. Despite these proven advantages, most persons with T1D engage in physical activity less frequently than their counterparts without the disease, and they may also lead unhealthy lifestyles that increase their risk of cardiometabolic disease [85]. When compared to regular daily activities, weight training and aerobic exercise have been shown in a few small trials to reduce the daily insulin demand [86]. Long-term exercise regimens have been shown to significantly improve whole-body insulin sensitivity (20–60%) while having little to moderate effects on hepatic insulin sensitivity [87,88]. Despite no improvements in HbA1c, six to twelve weeks of exercise training (cycling or aerobic training) have been demonstrated to enhance insulin sensitivity and lower daily insulin dosages [89].

### 3.3. Pharmacological Therapies Targeting Insulin Resistance in T1DM

At the moment of writing this literature review, there are no drugs approved to reduce IR in patients with T1DM. All the data related to pharmacological interventions targeting T1DM are data coming from exploratory studies or hypotheses regarding possible mechanisms linked to improvements in IR.

#### 3.3.1. Metformin

By activating AMP-activated protein kinase (AMPK) in the liver and skeletal muscle, a nutritional sensor that is active in conditions of low energy balance, metformin inhibits complex 1 of the mitochondrial respiratory chain and modifies the cellular energy state [6]. Reduced hepatic gluconeogenesis and hepatic glucose production are the main effects, but insulin-stimulated peripheral glucose uptake—particularly in skeletal muscle—also increases. Metformin further contributes to its glucose-lowering action by increasing intestinal production of GLP-1 and decreasing intestinal glucose absorption [90]. It has also been proposed that the gut microbiota’s composition has changed. Furthermore, metformin can improve endothelial function, inhibit the proinflammatory pathway in perivascular adipose tissue, inhibit STAT3 and monocyte-to-macrophage differentiation in vascular tissue, and reduce fatty acid oxidation and lipid-lowering through its pleiotropic effects, which are mediated by AMP-activated kinase [91].

In order to improve insulin sensitivity and glycemic control, restrict insulin dosage and weight gain, and perhaps lower cardiovascular risk over the long run, there has been an increasing interest in investigating the efficacy of metformin as an adjuvant to injectable insulin therapy in T1DM [92].

In a study that aimed to demonstrate that metformin therapy improves insulin sensitivity and vascular health in adolescents with T1DM, metformin was demonstrated to ameliorate insulin resistance despite baseline BMI, weight, fat mass, insulin dosage, and aortic and carotid health [93].

According to recent data, metformin lowers maximum carotid intima media thickness (cIMT) and LDL cholesterol and might extend the cardioprotective advantages in T1DM. Therefore, using metformin to lower insulin dosage requirements and improve cardiovascular risk management in T1DM may be justified. To determine if cardiovascular outcomes have indeed decreased, more outcome studies are required [6].

#### 3.3.2. Sodium–Glucose Cotransporter 2 Inhibitors (SGLT2is)

SGLT2is appear to be one of the potential treatments for double diabetes, particularly in people who are overweight and/or have cardiovascular risk factors. These agents work by blocking SGLT2, which lowers the renal plasma glucose threshold and causes glycosuria, consecutively reducing blood glucose. However, patients should be conscious of the risk of developing diabetic ketoacidosis, able to identify the symptoms, and gain the ability to act appropriately on their own [54,94].

The effectiveness of SGLT2i dapagliflozin as a supplement to insulin therapy was recently assessed in young Japanese T1DM participants who were overweight and had poor glycemic control despite intense insulin therapy. These subjects were diagnosed before the age of 15. The findings demonstrate the positive impact of adjunct dapagliflozin medication in young individuals with T1DM and support those found in earlier Caucasian research [94]. Throughout the trial period, there was a significant improvement in glycemic control, a significant drop in body weight and BMI, and a considerable reduction in insulin dosage [95].

#### 3.3.3. Glucagon-like Peptide-1 Receptor Agonists (GLP1-RAs)

GLP1-RAs are incretin medications that increase the production of glucose-dependent insulin, suppress the release of glucagon from pancreatic α cells in hyperglycemia states, diminish the stomach’s emptying, and affect appetite, resulting in a decrease in food consumption [96]. Interventions with liraglutide decreased HbA1c, daily insulin dosage, and body weight in patients with T1DM, according to the ADJUNCT studies; however, the risk of hypoglycemia and ketosis was also raised [97,98]. When a patient exhibits excess body weight, GLP1-RAs should nevertheless be taken into consideration.

#### 3.3.4. Thiazolidinediones

Because this therapeutic class increases insulin-dependent glucose disposal in skeletal muscle and adipose tissue and decreases hepatic glucose production, thiazolidinediones lead to improvements in insulin sensitivity and glucose control. Despite their effectiveness, their usage is limited by the secondary additional weight and fluid retention that is linked to cardiovascular risks [99,100].

## 4. Conclusions

Currently, the only validated methods to reduce insulin resistance in patients with T1DM are nutritional interventions and physical activity. Most of the mechanisms that are involved in the occurrence of IR in T2DM are also involved in the development of IR in T1DM. Currently, a set of therapies approved for the treatment of T2DM are studied as possible interventions in reducing IR in patients with T1DM; however, further research is needed in this direction.

## Figures and Tables

**Table 1:** Studies relating to insulin-resistant states in T1DM.

Authors	Study	Main Ideas
A M Arafat et al. [55]	The impact of insulin-independent, glucagon-induced suppression of total ghrelin on satiety in obesity in type 1 diabetes mellitus	in T1DM, glucagon-induced satiety was maintained, but not in obesity
Maria C Harpsøe et al. [57]	Body mass index and risk of autoimmune diseases: a study within the Danish National Birth Cohort	obesity as an insulin-resistance state was associated with a higher risk of type 1 diabetes
Mathilde Versini et al. [58]	Obesity in autoimmune disease: not a passive bystander	obesity is one of the main environmental factors influencing the development and course of autoimmune diseases the risk of developing T1DM is increased with higher infant birthweight (particularly >4000 g) insulin resistance is increased in obese persons with an adipokine profile of high resistin and low adiponectin
Daniel Vestberg et al. [60]	Relationship between overweight and obesity with hospitalization for heart failure in 20,985 patients with type 1 diabetes: a population-based study from the Swedish National Diabetes Registry	obesity as an insulin resistance state, especially severe obesity, is associated with hospitalization for heart failure in T1DM
Sarah A Price [61]	Obesity is associated with retinopathy and macrovascular disease in type 1 diabetes	BMI > 30 kg/m^2^ is the main risk factor for retinopathy and CVD despite similar HbA1 and the use of cardioprotective medications ∙
Orit Pinhas-Hamiel et al. [62]	Prevalence of overweight, obesity and metabolic syndrome components in children, adolescents and young adults with type 1 diabetes mellitus	women with T1DM were more likely to be overweight but not obese persons with T1DM who were overwieght/obese had higher risk of metabolic syndrome
Sarah K Holt et al. [63]	Prevalence of low testosterone and predisposing risk factors in men with type 1 diabetes mellitus: findings from the DCCT/EDIC	9.5% of T1DM patients had low testosterone levels, which is comparable to the overall population multivariate analysis that revealed a substantial correlation between low testosterone and obesity
Pawel Burchardt et al. [64]	Metformin added to intensive insulin therapy reduces plasma levels of glycated but not oxidized low-density lipoprotein in young patients with type 1 diabetes and obesity in comparison with insulin alone: a pilot study	after six months of treatment, those receiving metformin showed a substantial reduction in their A1C, BMI, glycated LDL, and triglyceride levels, but those receiving insulin therapy alone did not
S Vella et al. [65]	The use of metformin in type 1 diabetes: a systematic review of efficacy	metformin reduced insulin needs in these people, which is consistent with increased insulin sensitivity, and it also slightly reduced total cholesterol and LDL-C, according to one meta-analysis
Bo Ahrén et al. [66]	Efficacy and safety of liraglutide added to capped insulin treatment in subjects with type 1 diabetes: the ADJUNCT TWO randomized trial	body weight, HbA1c, and insulin needs were all decreased when liraglutide was added to capped insulin liraglutide increased the risk of hypoglycemia for the dose 1.2 mg and the risk of ketosis for 1.8 mg
Juan J Chaillarón et al. [51]	Estimated glucose disposal rate in assessment of the metabolic syndrome and microvascular complications in patients with type 1 diabetes	microvascular problems are linked to insulin resistance, which is prevalent in individuals with type 1 diabetes compared to other traditional factors like insulin needs, eGDR, as an indicator of insulin resistance, offers more valuable information
